# From Coalfields to Carbon Sinks: Examining the Policy Effects on the Dynamics of Ecosystem Services in the Watersheds of Eastern Kentucky, USA

**DOI:** 10.1007/s00267-026-02431-2

**Published:** 2026-04-09

**Authors:** Shreesha Pandeya, Buddhi R. Gyawali, Suraj Upadhaya, Maheteme Gebremedhin, Demetrio Zourarakis

**Affiliations:** College of Agriculture, Health and Natural Resources, Kentucky State University, Frankfort, KY USA

## Abstract

The Big Sandy River Basin (BSRB), which comprises the majority of the surface-mined and reclaimed areas, is a key restoration landscape in Eastern Kentucky. BSRB has a long history of coal mining, followed by various policies adopted to restore the post-mining ecosystems. Kentucky designates priority watersheds (PWs) within major river basins to address environmental issues and direct resources for focused implementation through coordinated efforts. However, clear watershed-scale evidence evaluating ecosystem service (ES) patterns in relation to these policy designations remains limited. Therefore, we conducted spatial and temporal mapping of carbon storage (CS) and sequestration (CSE) from 2001 to 2021 across selected PWs and NPWs, utilizing the National Land Cover Dataset (NLCD) and the InVEST model. The results revealed only a modest net increase in CS in both PWs (+0.93%) and NPWs (+0.16%) from 2001 to 2021. However, CSE patterns exhibited a trajectory towards recovery. Both PWs and NPWs experienced carbon loss during 2006-2011 and followed a gain afterwards until 2021. Between 2001 and 2021, CSE values were nearly 6.5 times higher in PWs (2.27 Mg C/ha) than in NPWs (0.35 Mg C/ha). The economic valuation (EV) of the CS revealed that the landscape offers climate-regulating ES worth more than 6100 USD/ha across years. This study utilized spatial statistics (Moran’s I test) that identified regions with high-high value and low-low value clusters and outliers of CSE across time. These findings support prioritization and monitoring in post-mining watersheds and provide an assessment framework for linking LULCC, ES, and watershed-level policy focus.

## Introduction

Global sustainability is now under serious threat as climate change, driven by anthropogenic activities and excessive greenhouse gas emissions, impacts both the planet and local communities. To reduce the impacts of such threats, especially carbon emissions, the world has been adopting various strategies at the local, regional, and global levels. Among numerous strategies, increasing the capacity of carbon storage in the terrestrial landscape plays a crucial role in reducing carbon dioxide (CO_2_) emissions in the atmosphere and thus helps combat the changing climate issue at a global scale (Min et al. 2025). Carbon storage in the landscape plays a significant role in the global carbon cycle, and it offers both natural and cost-effective solutions. Many national and international studies have focused their primary research and discussion topics on the impact of carbon emissions on the climate. However, the foundational focus is needed on understanding how terrestrial land use and land cover changes (LULCC) triggered by human activities have impacted the provision of ecosystem services (ES), thereby finding the solution to mitigate the large-scale emission of carbon in the atmosphere (Yang et al. 2024).

Nature provides numerous life-sustaining benefits, including clean air and water, fertile soil for food production, pollination, flood regulation, etc., which are collectively known as ecosystem services (ES) (U.S. Environmental Protection Agency 2024). These services include provisioning (PS), regulating (RS), supporting (SS), and cultural (CUS) services (Millennium Ecosystem Assessment 2005). Among these, climate regulation falls within the “regulating ecosystem services” that play a significant role in mitigating climate change by absorbing greenhouse gases (GHGs). Ecosystem services are vital for the environment, human well-being, and economic growth, yet they are limited and often overlooked (U.S. Environmental Protection Agency 2024). They form the foundation of Earth’s life-support systems, directly and indirectly benefiting people and contributing significant economic value (Costanza et al. 1998).

Despite their importance, ecosystem services are significantly influenced by anthropogenic activities, including land use and land cover change (LULCC) (Chang et al. 2022; Demissie 2022; Houghton 2012; L. Li et al. 2023; Z. Li et al. 2004; Wang et al. 2024). Various social, economic, natural, and policy factors drive LULCC, and among these drivers, surface mining for coal extraction remains one of the most intensive land use practices (Höök & Aleklett 2009; Zégre et al. 2013). Surface coal mining is the process of extracting minerals near the Earth’s surface through a series of activities, including vegetation clearing, topsoil removal, drilling, and blasting the hard strata over the coal seams, and then extracting the coal (Balasubramanian 2016). The central Appalachian Mountains in the United States, particularly in northeastern Tennessee, eastern Kentucky, southwestern Virginia, and southern West Virginia, have experienced extensive surface coal mining, notably mountaintop removal (Wickham et al. 2014). This long history of coal extraction has substantially altered ecosystem services, resulting in forest loss, soil degradation, increased landslides and floods, water contamination, and the conversion of carbon sinks to sources across the Appalachian region (Kandel et al. 2023).

The Big Sandy River Basin (BSRB) is a part of the central Appalachian mountainous region in eastern Kentucky. The BSRB is characterized by extensive forest cover and wetlands that have historically functioned as a significant carbon sink and offer various ecosystem services (Liu et al. 2006). However, the legacy of surface mining, combined with extensive clearing of forested lands for mining-related activities, has severely altered the ecological functions and the provision of ES, including carbon storage and sequestration capacity of the land (Cribari et al. 2022).

In the context of BSRB, mining activities and the use of coal for energy production serve as the two major sources of carbon dioxide (CO_2_) emissions in the atmosphere. In addition, surface mining practices for coal extraction in the Appalachian mountains further weaken the ecosystem structure and contribute to additional emissions of CO_2_ through the complete removal of the native vegetation (Amichev et al. 2008). Beyond the irreversible alteration of the landscape associated with mountaintop removal and surface mining, waste disposal into the valleys led to the burial of headwater streams, thereby polluting water bodies within the watersheds and disrupting ecosystem services further. These changes have directly affected wildlife and their habitat (Christopher et al. 2020) as well as the local communities living in those areas and downstream (Lindberg et al. 2011). Given the adverse effects on the environment and public health, the majority of the local communities are now actively engaged in non-governmental and governmental restoration efforts targeted at improving the ecosystem services in previously mined areas, and are hoping to regain the beauty and health of the ecosystem as it was before the mining activities occurred (Poudyal et al. 2024).

Previously mined areas have undergone extensive reclamation efforts after the establishment of regulatory frameworks under the Surface Mining Control and Reclamation Act (SMCRA) of 1977. Although SMCRA promoted reclamation, outcomes were limited because mining operators often planted fast-growing invasive species that worsened soil conditions and degraded ecosystem services (X. Li et al. 2018). To address these issues, the Appalachian Regional Reforestation Initiative (ARRI) was launched in 2004, introducing the Forest Reclamation Approach (FRA) to improve reforestation on abandoned and post-1977 mine sites. While these efforts have achieved moderate success, reclaimed lands in BSRB continue to face challenges such as compacted soils, reduced infiltration, and higher runoff, which restrict full restoration of ecosystem services to pre-mining conditions (Butler et al. 2015; Li et al. 2018). In addition, pressures from urbanization, energy, and economic development further limit the restoration of the landscape needed for enhancing carbon storage (CS) and sequestration (CSE) capacity. As such, there exists a clear trade-off between economic development and the ecological functions required for sustaining the needed ecosystem services (Bai et al. 2021).

To support an integrated watershed management framework, the Kentucky Division of Water (KDOW), following EPA guidelines, designated certain watersheds within Kentucky’s river basins as priority watersheds (PWs). The PWs are designated through a policy-based selection process that considers the vulnerability scores, the need for action, and the feasibility of intervention. A priority watershed serves as a strategy to coordinate and target resources for improving water quality (EEC 2021). It considers both existing problems and the area’s capacity, such as community interest, active groups, and potential partners. Because PWs receive more attention for funding and resources, management efforts are mainly directed toward them. To date, no study has compared carbon storage (CS) and sequestration (CSE) between priority (PWs) and non-priority watersheds (NPWs) of BSRB, especially at a spatial scale useful for watershed planning. In this study, CS refers to the amount of carbon stored in the vegetation, soil, and biomass within the landscape as determined by the land use and land cover (LULC) types. In contrast, the difference in stored carbon between present and future land use scenarios determines the total sequestered carbon for each pixel, defining CSE. There is also a need to identify watersheds that are vulnerable, recovering, or have the potential for recovery to develop a plan for integrated land management strategies at a watershed scale. Such strategies aim to optimize CS and CSE capacity while supporting sustainable development in BSRB. Therefore, this study focuses on comparing the spatial and temporal dynamics of carbon storage and sequestration between PWs and NPWs and highlighting the importance of conserving natural assets based on their calculated economic values, utilizing GIS and remote sensing techniques.

## Materials and Methods

### Study Area

**Fig. 1:**
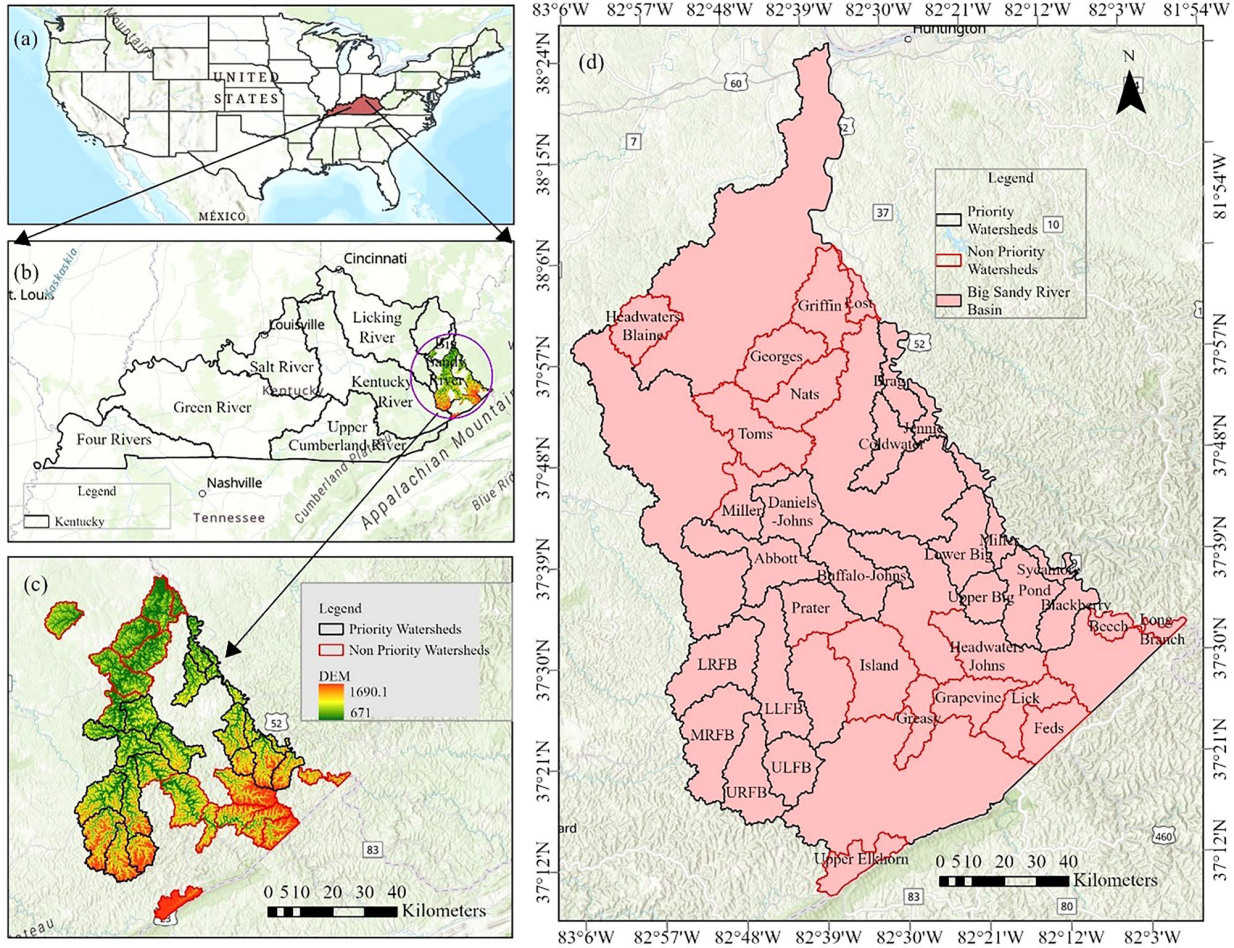
Study area showing (**d**) selected watersheds (PWs and NPWs) within (**b**) Big Sandy River Basin of (**a**) Kentucky and (**c**) elevation (meters)

The BSRB is located in the Eastern Kentucky Coal Field physiographic region, exhibiting complex topography (Carey 2009) (Fig. 1). The coordinates of BSRB range from a latitude of 37.5 to 38.5 degrees N and a longitude of −83 to −81.5 degrees W, and the slope ranges from 0 to 72 degrees. The study area includes watersheds within four HUC-8 watersheds, namely, Big Sandy, Lower Levisa, Upper Levisa, and Tug Fork. The PWs and NPWs, which represent the study area within the BSRB, are Hydrologic Unit Code (HUC) -12 watersheds. There are 17 PWs in the BSRB (Fig. S1). Furthermore, a total of 17 watersheds, which are either adjacent to PWs or those that are of particular interest to the basin managers in the coming 3-5 years, are selected as NPWs (Fig. S2). These 17 PWs and 17 NPWs lie entirely within BSRB and cover Martin, Pike, Floyd, and Lawrence Counties. We conducted this study within BSRB because basins represent open and interconnected systems where the ecological, socio-economic, and cultural components are strongly linked. River Basin serves as the fundamental unit of an ecosystem in its entirety, specifically in the context of national-level spatial planning (S. Yang et al. 2024).

### Data Preparation

We downloaded boundaries of the watersheds (HUC-8) and sub-watersheds (HUC-12) from the National Hydrography Dataset (https://nhd.usgs.gov). The specific PWs and NPWs were selected from the downloaded data and extracted as the study area shapefile. The analysis of spatial and temporal dynamics of CS and CSE was performed using the InVEST 3.16.1 model (https://naturalcapitalproject.stanford.edu/software/invest/invest-downloads-data). The land use and land cover (LULC) maps, which are the primary input of the model, were gathered for 2001, 2006, 2011, 2016, and 2021 from Pandeya et al. (2025). The reclassified LULC map featured a land cover distribution for eight LULC classes: water, developed, barren, forests, shrubland, herbaceous, pasture/cultivated, and wetlands, based on the National Land Cover Dataset (NLCD) at a 30-m spatial resolution. The detailed mapping of trends and patterns of spatial agglomeration was conducted at the census block group (CBG) level. The CBG boundary was downloaded from the United States Census Bureau (https://www.census.gov/geographies/mapping-files/time-series/geo/tiger-line-file.html).

### Data Analysis

#### Climate Regulating Ecosystem Services

For mapping climate-regulating ecosystem services, we used the carbon storage and sequestration model in the InVEST software. This model estimates the amount of carbon stored (metric tons) in each pixel in the landscape (Natural Capital Project, 2019). It requires LULC maps and four carbon pool coefficient values: aboveground biomass (AB), belowground biomass (BB), soil organic matter (SOM), and dead organic matter (DOM). As the reliability of the InVEST outputs depends on the quality of the inputs (Hamel et al. 2021), we used LULC maps that were assessed for accuracy and validated against field data (Pandeya et al. 2025). This process ensured a reliable representation of LULC dynamics in the study area. The carbon storage and sequestration model provides spatial information on the location and amount of carbon stored in a landscape. At least one coefficient of carbon pools or carbon density (Mg C/ha) is needed for estimation. Coefficient values for AB, BB, SOM, and DOM were compiled from various studies (Abbasnezhad et al. 2024; Amichev et al. 2008; Bai et al. 2021, 2024; Benez-Secanho et al. 2022; Benez-Secanho & Dwivedi 2020; Brown et al. 1999; Geneletti et al. 2018; Gurung et al. 2018; Heath et al. 2011; IPCC 2006; Jerath et al. 2016; X. Li et al. 2018; Olson et al. 1985; Qiu & Turner 2013; Sharp et al. 2014; Silver et al. 2010; Timilsina et al. 2013; Waleed et al. 2024). The applied input coefficients (Table S1) represent the average of these sources, which was then used to estimate carbon storage per pixel (Benez-Secanho et al. 2022). The model produced outputs, including tabular results and raster (TIFF) images, which represent total carbon stored per pixel (Mg C) at a spatial resolution of 30 m. Those outputs were exported from the InVEST database to ArcGIS Pro for further analysis and mapping. Since each pixel corresponds to an area of 0.09 ha, pixel-level carbon storage and sequestration values for all years were converted to carbon density (Mg C/ha) by dividing carbon per pixel by the pixel area using Raster Calculator. These density values were used to generate spatial maps/figures of carbon storage (CS) and carbon sequestration (CSE). The total absolute values generated by InVEST model are reported in tables in units of megagrams of carbon (Mg C). They represent the sum of carbon across all the pixels within each watershed group. Next, these total carbon storage and sequestration values were used to calculate area-weighted mean carbon density (Mg C/ha) that shows watershed-level trends and patterns. This was calculated by dividing the total carbon values (Mg C) by the corresponding area (ha) of PWs and NPWs. The rate of change in the CS and CSE values in percentage was calculated to show the relative trends and patterns of change.

In addition, we evaluated statistical differences in watershed-level normalized carbon storage and sequestration values between 17 PWs and 17 NPWs. The Shapiro-Wilk test was applied to check the normality of CS and CSE distributions. This test is suitable for moderate sample sizes (*n* < 50) (Mishra et al. 2019). The data exhibited non-normal or mixed distributions with *p* < 0.05. Therefore, a two-sided Mann-Whitney U test was selected to evaluate statistical differences in CS and CSE values between selected PWs and NPWs, at a significance level of p = 0.05. All statistical analyses were performed using Python version 3.13.1.

Furthermore, to account for the variability in carbon pool coefficients used by the InVEST model, we conducted an uncertainty analysis using the minimum and maximum values of carbon coefficients. We used these values to generate low and high carbon pool coefficient tables, in addition to the average table used in the main analysis. We ran the InVEST model for the years 2001 and 2021 for both PWs and NPWs. The midpoint between the low and high estimates represents the mean, and half of the difference between the low and high estimates provides the uncertainty margin. This approach enabled us to quantify the sensitivity of total carbon storage and sequestration estimates to variations in carbon density parameters.

#### Economic Valuation of Ecosystem Services

We measured the monetary value of CS and CSE at the pixel level using the InVEST model. Zero economic value was assigned to urban pixels, thereby considering only the natural areas for ES valuation (Moore et al. 2011). For this, the required input is the social cost of carbon (SCC), which determines the economic value linked with carbon storage in the landscape. The SCC represents the economic damage incurred due to the emissions of carbon to the environment (Abbasnezhad et al. 2024; Benez-Secanho & Dwivedi 2020; Greenstone et al. 2013). The average values of SCC from several similar studies are considered for analyzing the economic value of ES (Costanza et al. 1998; Li et al. 2018; Rosenberger & Loomis, 2003). The study conducted by Atkinson & Gundimeda (2006) used SCC value at 23$/ t C with a reasonable range between $5.5/t C and $46/t C according to 2015 US$. In 2010, the U.S. government’s Interagency Working Group on SCC reported a range of $5.7/t C to $73.6/t C according to 2015 US$, and it recommended an average value of $23.8/t C for the regulatory impact analysis. The Chicago Climate Exchange (CCX) provides an average price of $7.7/t C with a historical range from $0.2/t C to $24.11/t C (2015 USD $) (Moore et al. 2011). The study of (Abbasnezhad et al. 2024; Benez-Secanho & Dwivedi 2020) used USD $7.26/t C/year (2020 USD $). Another study (X. Li et al. 2018) used an average of $24/t C/ year for the value of carbon sequestered. In the study, $26/t C/year was derived from literature, and this constant dollar unit value was applied to show the biophysical estimates of carbon storage and sequestration in economic terms for the years 2001, 2006, 2011, 2016, and 2021. Although this approach does not reflect changing market dynamics or inflation, it enabled spatial and temporal comparison of ecosystem services dynamics in the study area. Finally, the economic values associated with the LULC types were extracted from the InVEST database and taken into Excel 2021 for further analysis and representation in tables. The raster map imported from InVEST into ArcGIS Pro had a monetary value assigned to each pixel within the watersheds. Positive values represented the benefit or gain in carbon sequestration, while negative values represented the expense or loss of carbon emissions (Sharp et al. 2014).

#### Spatial Statistical Analysis

The spatial patterns of CS and CSE were further examined independently in ArcGIS Pro software 3.2.1, using Global and Local Moran’s I statistics to evaluate spatial autocorrelation and clustering. We used the percentage change in CSE at the CBG level to compare PWs and NPWs and applied a Spatial Autocorrelation test. Since our goal was to see how and where carbon storage change patterns had shifted, the test was focused on CSE. Spatial autocorrelation is crucial in modeling spatial data, and Moran’s I is a widely used statistic among geographers and researchers for this purpose. Global Moran’s I must first confirm clustering before running localized tests (Shobairi et al. 2024). Therefore, following the principle, we conducted the Global Moran’s I test followed by the Local Moran’s I test.

Global Moran’s I measures the spatial autocorrelation of CS and CSE values within a watershed (ESRI 2025). The null hypothesis assumes that the CS and CSE (Mg/ha) in the PWs and NPWs are randomly distributed. The index ranges from −1 to 1: values greater than 0 indicate positive spatial autocorrelation (clustering), values less than 0 indicate negative spatial autocorrelation (dispersion), and a value of 0 indicates randomness (Akrofi 2024; ESRI 2025; Muga et al. 2025; Yang et al. 2024). The Local Moran’s I determines the spatial location of the center of agglomeration, revealing the spatial clusters (H-H cluster or Hot Spots/L-L clusters or Cold Spots) and outliers within the study area, thereby providing a detailed localized level of analysis (Muga et al. 2025; X. Yang et al. 2024). ArcGIS Pro follows a permutation-based test with 95% confidence interval for determining the statistical significance of spatial clustering.

## Results

### Spatial and Temporal Changes of Carbon Storage from 2001 to 2021

In PWs, total CS values varied from 37.25 × 10⁶ Mg C in 2001 to 37.60 × 10⁶ Mg C in 2021, while in NPWs, CS ranged from 33.94 ×  10⁶ in 2001 to 33.99 × 10⁶ Mg C in 2021 (Supplementary Table S2). The area-weighted mean carbon storage in PWs ranged from 241.5 Mg C/ha in 2001 to 243.8 Mg C/ha in 2021, while in NPWs it ranged from 237.7 Mg C/ha in 2001 to 238.1 Mg C/ha in 2021 (Table 1).

**Table 1:** Area-weighted mean carbon storage (CS) and associated economic values at the watershed level from 2001 to 2021

Year	CS in PWs (Mg C/ha)	Total Economic Value in PWs (USD/ ha)	CS in NPWs (Mg C/ha)	Total Economic Value in NPWs (USD/ha)
2001	241.5	6278.2	237.7	6179.7
2006	238.7	6205.2	234.5	6094.8
2011	238.2	6192.6	233.9	6080.4
2016	238.4	6231.9	234.9	6103.4
2021	243.8	6340.2	238.1	6191.3

**Fig. 2:**
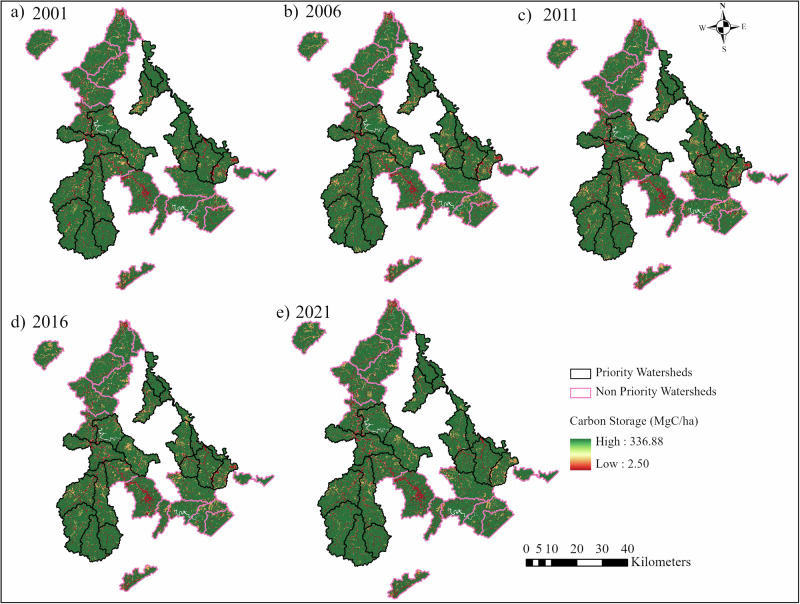
Spatial and temporal distribution of carbon storage (CS) in priority watersheds (PWs) and non-priority watersheds (NPWs) in 2001 (**a**), 2006 (**b**), 2011 (**c**), 2016 (**d**), and 2021 (**e**)

From 2001 to 2021, CS increased by approximately 0.93% in PWs and 0.16% in NPWs. We observed a decrease in CS between 2001 and 2006 in both PWs (−1.20%) and NPWs (−1.36%), but CS increased between 2011 and 2021 in both PWs (2.65%) and NPWs (1.52%) (Supplementary Table S3). This revealed that relative gains in carbon storage over time were higher in PWs than in NPWs. However, the differences in carbon storage values at the watershed level were found to be statistically non-significant (*p* > 0.05) between PWs and NPWs across all study years (Supplementary Table S4). The spatial and temporal changes of CS for 2001, 2006, 2011, 2016, and 2021 are shown in Fig. 2. It shows pixel-level carbon storage density (Mg C/ha) at 30 m spatial resolution, representing spatial variability across the landscape. High CS (green) corresponds to forest cover and wetlands, moderate CS (yellow) is associated with shrublands, herbaceous, and pasture lands, while low CS (red) occurs in barren land, water, and developed LULC types.

Furthermore, the uncertainty analysis indicated that carbon storage estimates were influenced by the range of carbon pool coefficients used in the InVEST model, as shown in Supplementary Table S5. In the PWs, total carbon storage in 2001 varied from 8.53 × 10⁶ to 76.84 × 10⁶ Mg C, corresponding to a mean estimate of 42.68 ± 34.15 × 10⁶ Mg C. In 2021, the range was 8.67 × 10⁶ to 77.39 × 10⁶ Mg C (mean: 43.03 ± 34.36 × 10⁶ Mg C). Similar patterns were observed in the NPWs, where carbon storage varied from 7.80 × 10⁶ to 70.06 × 10⁶ Mg C in 2001 (mean: 38.93 ± 31.13 × 10⁶ Mg C) and from 7.87 × 10⁶ to 70.04 × 10⁶ Mg C in 2021 (mean: 38.95 ± 31.08 × 10⁶ Mg C). Although absolute values differed across the low and high-parameter sets, the relative differences between PWs and NPWs, as well as the overall temporal patterns, remained consistent.

### Spatial and Temporal Changes of Carbon Sequestration from 2001 to 2021

The CSE values varied from a lowest of −0.45 × 10^6^ Mg C to the highest of 0.63 × 10^6^ Mg C in PWs and from −0.46 × 10^6^ to 0.45 × 10⁶ Mg C in NPWs (Supplementary Table S6). Between 2001 and 2021, the area-weighted mean carbon sequestration was 2.27 Mg C/ha in PWs and 0.35 Mg C/ha in NPWs (Table 2). This indicated nearly 6.5 times higher carbon sequestration in PWs. The temporal analysis highlighted a period of carbon loss from 2001 to 2011, followed by an increasing trend of CSE from 2011 to 2021 in both PWs and NPWs. From 2016 to 2021, the total carbon sequestration was the highest, i.e., 4.08 Mg C/ha in PWs and 3.15 Mg C/ha in NPWs (Table 2), indicating approximately 1.3-fold higher CSE in PWs than NPWs.

**Table 2:** Area-weighted mean carbon sequestration (CSE) and associated economic values at the watershed level across different time periods from 2001 to 2021

Year	CSE in PWs (Mg C/ha)	Economic Value in PWs (USD/ha)	CSE in NPWs (Mg C/ha)	Economic Value in NPWs (USD/ha)
2001–2006	−2.92	−67.30	−3.22	−75.14
2006–2011	−0.52	−12.06	−0.49	−12.47
2011–2016	1.56	36.37	0.91	22.27
2016–2021	4.08	95.37	3.15	74.23
2001–2021	2.27	35.59	0.35	5.95

**Fig. 3:**
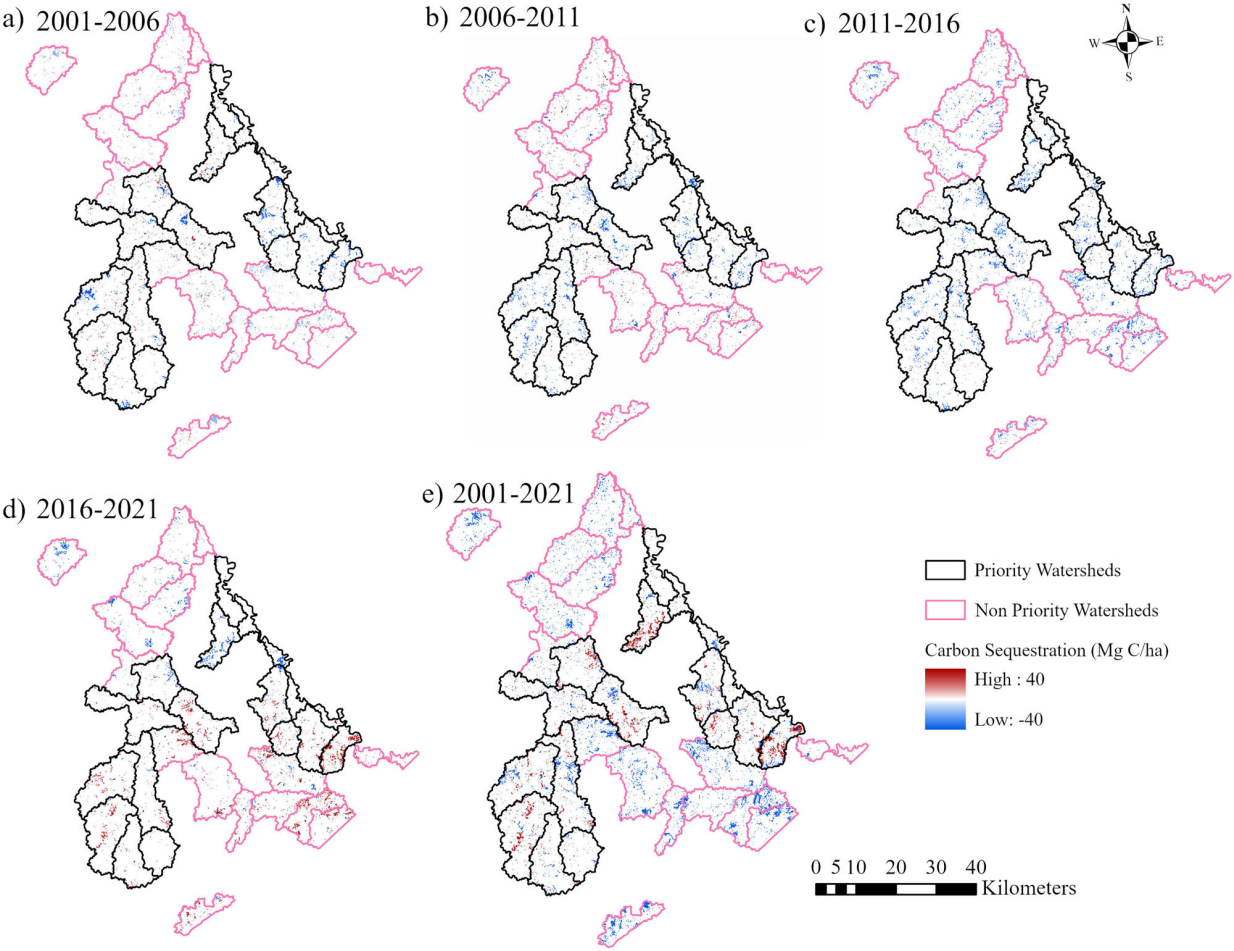
Spatial and temporal distribution of carbon sequestration (CSE) in PWs and NPWs between 2001-2006 (**a**), 2006-2011 (**b**), 2011-2016 (**c**), 2016-2021 (**d**), and 2001-2021 (**e**)

In NPWs, we observed a comparatively higher rate of carbon loss. Although PWs experienced a period of some declines in CSE, these declines were counterbalanced by subsequent periods of recovery, highlighting the ecosystem resilience. In contrast, the NPWs exhibited greater instability, with some periods having negative sequestration values of carbon (Fig. 3). The carbon sequestration values are expressed in Mg C/ha at the pixel resolution, where positive values (red) indicate carbon gain and negative values (blue) indicate carbon loss. Areas with white symbology represent locations with near-zero change during the corresponding interval. The greater changes in CSE occurred in areas with high negative or positive values of area percentage change of LULC, which were seen in the first two time intervals in both PWs and NPWs (between 2001 and 2006, and between 2006 and 2011). However, the results from the statistical test revealed that there was no statistically significant difference (*p* > 0.05) in CSE values between the PWs and NPWs across time intervals (Supplementary Table S7).

The uncertainty analysis demonstrated that sequestration estimates reflected the variability introduced by carbon pool coefficients. In the PWs, carbon sequestration between 2001 and 2021 ranged from 0.14 × 10^6^ to 0.55 × 10^6^ Mg C, with a mean value of 0.35 ± 0.21 × 10⁶ Mg C. In the NPWs, sequestration ranged from −0.02 × 10^6^ to 0.07 × 10^6^ Mg C, with a mean value of 0.05 ± 0.05 × 10^6^ Mg C. Despite these ranges of absolute values, the relative differences between PWs and NPWs were found to be consistent across the parameters used.

### Economic Valuation

The total economic value (EV) of carbon storage increased from 6278.18 USD/ha to 6340.17 USD/ha in PWs and from 6179.7 USD/ha to 6191.3 USD/ha in NPWs from 2001 to 2021 (Table 1). The overall EV exhibited an increasing trend from 2001 to 2021; however, the decline in the EV occurred between 2006 and 2011. The overall increase in the EV from 2001 to 2021 in the NPWs (0.16%) was still lower than the PWs (0.93%). Similarly, the decline in EV from 2006 to 2011 was found to be relatively higher in NPWs (−1.59%) than in PWs (−1.42%).

**Fig. 4:**
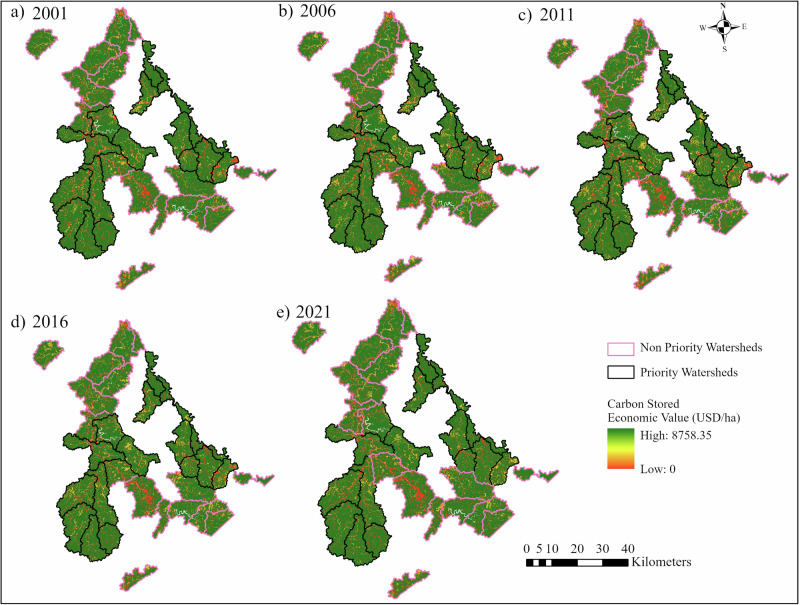
Spatial and temporal distribution of economic value (USD/ha) of carbon storage in PWs and NPWs in 2001 (**a**), 2006 (**b**), 2011 (**c**), 2016 (**d**), and 2021 (**e**)

**Fig. 5:**
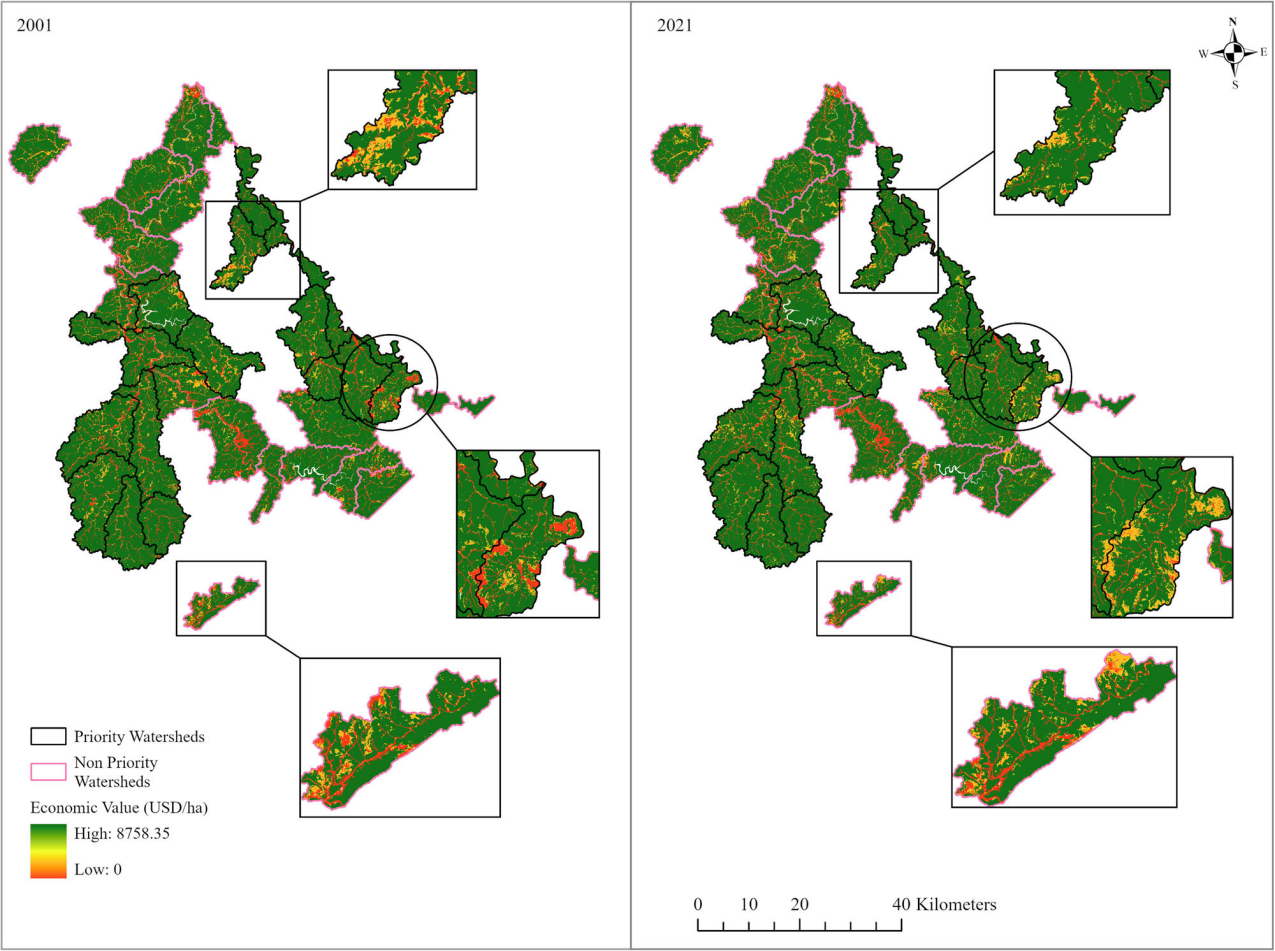
Early and later-period scenario of economic valuation (EV) distribution maps in 2001 and 2021 in PWs and NPWs

Spatially, the EV maps (Fig. 4) show the economic value distribution across the study area. The green symbology represents high EV, the yellow represents moderate, and the red represents low EV. The legend in the figure shows the EV across the LULC types at pixel resolution, which means some pixels have 0 USD/ha, and some pixels have an EV as high as 8758.35 USD/ha, corresponding to the carbon values with the type of LULC existing at that pixel. The areas with notable differences between the early year (2001) and the latest year (2021) are shown in Fig. 5, which highlights the early (2001) and later-period (2021) scenario of EV distribution at the spatial scale. We observed that the EV has increased in some watersheds like Buffalo Creek-John’s Creek (PWs) and Upper Elkhorn Creek (NPWs) (Fig. 5).

### Spatial Statistical Analysis

Global Moran’s I test for CS revealed that the distribution of CS values across both PWs and NPWs is significantly clustered for the years 2001, 2006, 2011, 2016, and 2021, with a *p* < 0.05 (Supplementary Table S8). However, CSE exhibited significant spatial clustering (*p* < 0.05) only between 2001 and 2006 (Supplementary Table S9). This suggests that there was an increase in spatial randomness over time in terms of carbon sequestration.

**Fig. 6:**
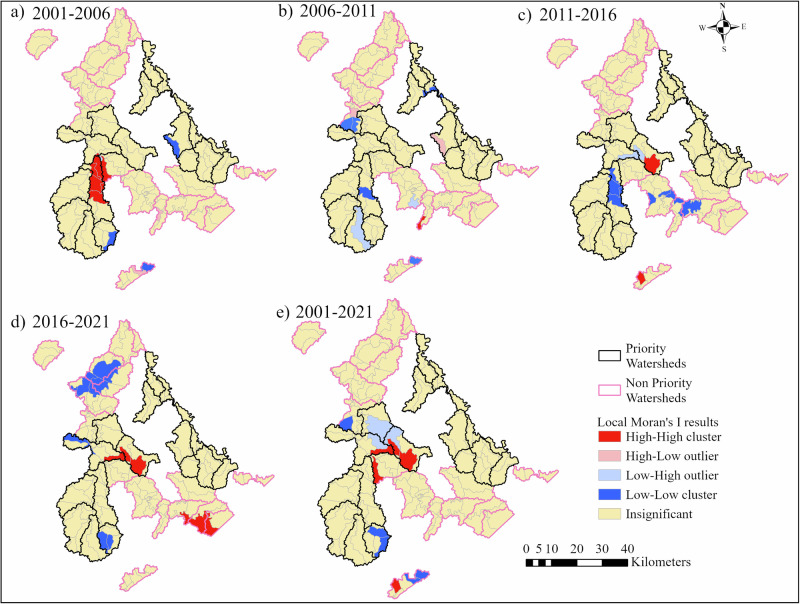
Local Moran’s I analysis showing clusters and outliers between 2001-2006 (**a**), 2006-2011 (**b**), 2011-2016 (**c**), 2016-2021 (**d**), and 2001-2021 (**e**) in PWs and NPWs

Local Moran’s I identified five types of spatial autocorrelation: High-High clusters (H-H), Low-Low Clusters (L-L), High-Low outliers (H-L), Low-High outliers (L-H), and insignificant (Fig. 6). Between 2001 and 2006, H-H clusters were mainly located in the southwestern PWs and the western part of NPWs, specifically in the Lower Left Fork-Beaver Creek (PWs) in Floyd County and Prater Creek (NPWs) of the Levisa Fork watershed. L-L clusters were found in the Lower Big Creek (PWs) in Martin County and Upper Left Fork Beaver Creek (PWs), while in NPWs, the upper Elkhorn Creek has a mixed region of L-L, H-L, and insignificant values. Between 2006 and 2011, the H-H clusters decreased while the L-L clusters significantly increased in new locations; however, with the Local Moran’s I value of −0.06 in PWs and a reduced value of 0.013 in NPWs (Supplementary Table S10), this indicated a weak spatial autocorrelation. L-L clusters were seen in the Lower Left Fork of Beaver Creek (PWs) in Floyd County, Jennie Creek (PWs) in Martin County, and Miller Creek (NPWs). Between 2011 and 2016, a notable clustering of high-high (H-H) values was observed in Buffalo Creek (PWs) and Upper Elkhorn Creek (NPWs). At the same time, a low-low (L-L) cluster expanded in the Lower Left Fork of Beaver Creek (PWs), as well as in Island, Grapevine, and Greasy Creek (NPWs). This pattern represents a significant change compared to the period between 2006 and 2011. Between 2016 and 2021, the H-H clusters expanded significantly in the Buffalo Creek (PWs) and southeastern region of BSRB in NPWs, namely Lick, Feds, and parts of Grapevine Creek. L-L clusters remained persistent in the Lower Left Fork-Beaver Creek (PWs), while in NPWs, L-L clusters expanded broadly towards the northeastern part of the BSRB, namely Toms, Nats, and Georges Creek along Levisa Fork. Overall, in PWs, between 2001 and 2021, the parts of Abbot Creek and Daniel’s Creek of Levisa Fork stood as L-H outliers. In the NPWS, between 2001 and 2021, we did not identify any significant outliers. However, H-H clusters were found in the Prater Creek (NPWs) and Upper Elkhorn Creek (NPWs). L-L clusters were found in the Miller Creek and the eastern part of Upper Elkhorn Creek.

## Discussion

**Fig. 7:**
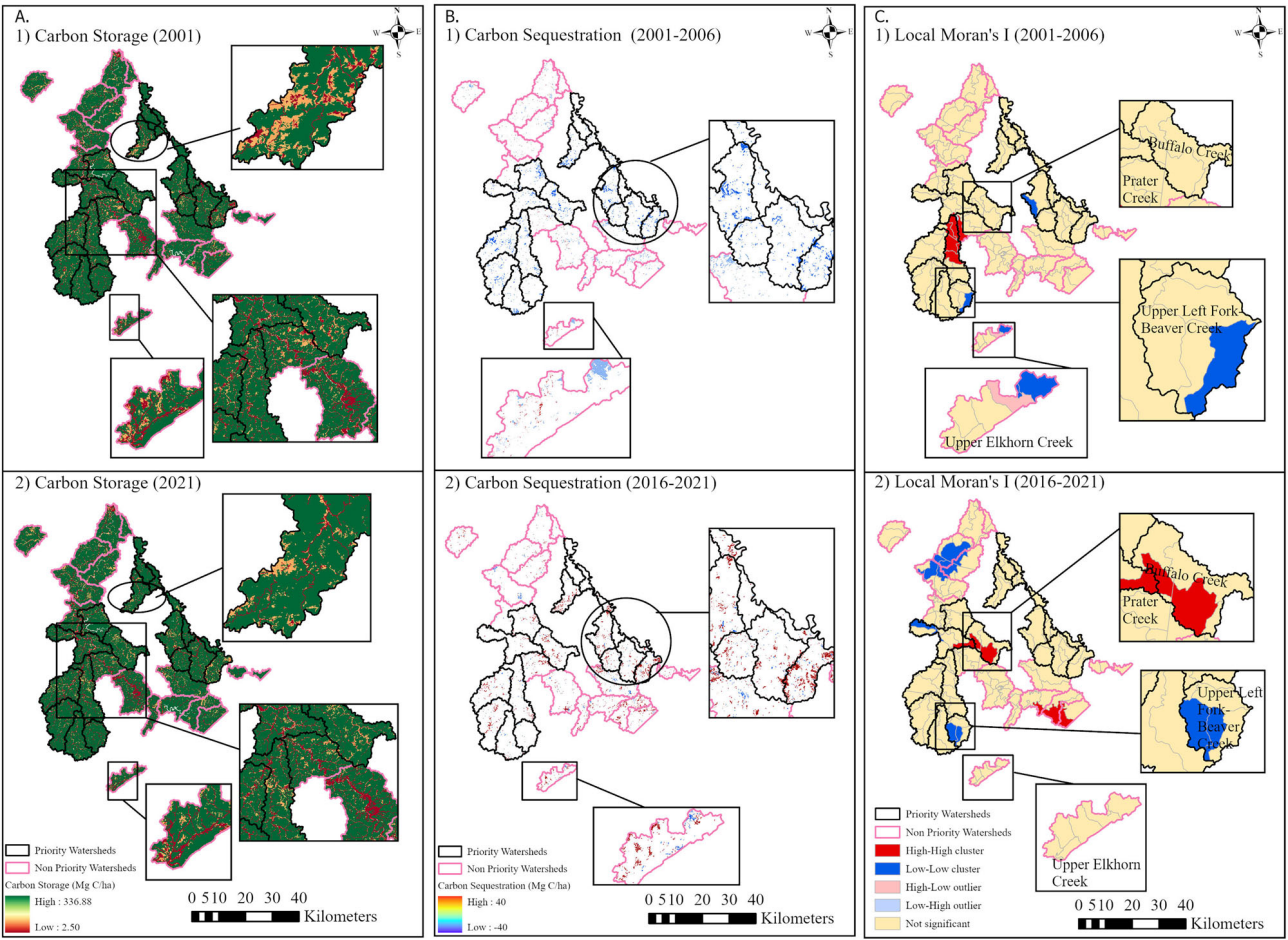
Integrated map showing the before (1) and after (2) scenarios of spatial-temporal variations in Carbon storage (**A**); Carbon sequestration (**B**), and Local Moran’s I (**C**) in PWs and NPWs

This study demonstrates that climate-regulating ecosystem services (ES) in the Big Sandy River Basin (BSRB) followed a clear disturbance-transition-recovery trajectory between 2001 and 2021, which could have been influenced by LULCC, reclamation practices, and watershed prioritization policies. The ecosystem services fluctuated in both PWs and NPWs throughout the two decades, where CS and CSE values were found to be slightly higher in PWs than in NPWs across all years. However, the differences were statistically nonsignificant. This finding illustrates a comparable distribution pattern of measured ecosystem services between PWS and NPWs, although spatial patterns suggest a relatively visually stronger recovery trend in PWs, particularly between 2016 and 2021. CS remained spatially clustered throughout the two decades, whereas CSE showed more dynamic spatio-temporal variation. The economic valuation revealed that BSRB, comprising both PWs and NPWs, is worth more than 6100 USD/ha (Table 1), which signifies that BSRB plays a vital role in climate regulation and has the potential to offer substantial environmental and economic benefits to the local communities. To further illustrate clarity, Fig. 7 shows an integrated before-and-after comparison of carbon storage and sequestration, and Local Moran’s I result. The map shows changes that suggest progressive improvement in climate-regulating ecosystem services in PWs (mainly Buffalo Creek) and are consistent with the broader patterns observed across the watersheds. However, recovery patterns remain spatially heterogeneous, with some watersheds continuing to exhibit L-L clusters of carbon sequestration. These indicate the persistent vulnerability and the need for continued monitoring and targeted restoration.

The spatial distribution patterns of CS are mainly attributed to the extensive forest cover characterizing eastern Kentucky, which accounts for 87% and 84% of the PWs’ and NPWs’ total land area, respectively. Forests and dense vegetation play a crucial role in CS and CSE, providing ES like climate regulation, flood and landslide reduction, preserving soil structures, and providing welfare to the human population and wildlife. Between 2001 and 2021, the climate-regulating ES showed a decline (2001–2006), a further decline (2006–2011), and a subsequent recovery (2011–2021), leading to the moderate stabilization of the ES gain. The early decline (2001–2006) coincided with the period before implementation of the forest reclamation approach (FRA) under ARRI, when reclamation relied on fast-growing or invasive species that contributed little to the recovery of ecosystem services. Continued decline between 2006 and 2011 reflects the transitional phase in which earlier vegetation stands were removed to implement FRA-guided native hardwood forest restoration. These efforts temporarily reduced CS and CSE but were necessary for establishing native forest systems capable of long-term ES restoration. The recovery observed after 2011 and until 2021 reflects the emerging benefits of FRA-aligned restoration, which is expected to enhance carbon storage and ecological stability over time.

The increased developed land cover and slower forest stabilization in NPWs compared to PWs (Pandeya et al. 2025), and LULCC fluctuations linked to socio-economic and development factors could potentially produce variations in CS and CSE (Xie et al. 2023). This result is consistent with the findings of Parsa et al. (2016), which highlight that forests decline at the expense of increased barren lands and agricultural/pasture lands. Areas with high CS corresponded to areas dominated by forests, wetlands, grasslands/herbaceous, and cultivated/pasture LULC types, while areas with low CS were concentrated in the urban landscape dominated by construction/developed LULC and barren lands, consistent with findings of Ma et al. (2025) and Sun et al. (2023). The major decline in CSE between 2006 and 2011, consistent with the results of LULCC presented in Pandeya et al. (2025), where forest and shrubland areas declined, highlighting a critical relationship between the LULCC and ES. Reclamation activities in the coal-mined areas and a watershed prioritization framework for management could have supported the restoration patterns of ecosystem services as observed in PWs. Other factors like soil conditions and species selection for reclamation may have played an important role in determining the rates of CSE by the forests and the overall success of restoring ES (Li et al. 2018). Similarly, in addition to policy interventions, factors such as passive regeneration on abandoned mine lands or a decrease in coal extraction linked to economic transitions in the Appalachian coalfields may also have contributed to CS and CSE dynamics over time.

The findings from this study identified several areas that require targeted conservation efforts. The regions where H-H and L-L clusters are observed remain consistent with previous studies of Liu et al. (2014), Shobairi et al. (2024), and Were et al. (2016), where H-H clusters are typically found in protected regions, mainly forested areas, while L-L clusters are pronounced in urbanized and barren/mined lands. In BSRB, significant H-H clusters were more prevalent in PWs, whereas L-L clusters were more evident across NPWs. In NPWs, Upper Elkhorn Creek and Prater Creek-Levisa Fork emerged as highly vulnerable watersheds due to fluctuating CSE values associated with unstable LULCC patterns. The vulnerability of Upper Elkhorn Creek could have been further reinforced by the recent flooding of 2022, which hit the city of Jenkins. In PWs, Upper Left Fork Beaver Creek exhibited greater sensitivity to LULCC and the corresponding altered CSE values, whereas Buffalo Creek demonstrated strong and increasing carbon sequestration patterns. This difference could be attributed to several factors, such as conservation efforts, land use policies, reclamation initiatives, etc. (Atalay 2025). However, these watersheds require deeper causal investigations to identify the drivers influencing these spatial-temporal variations in ecosystem services performance.

Despite the collective effort of the government, non-governmental organizations, and local communities to reclaim the landscape and restore ecosystem services to the pre-mining stage, the success is moderate (Poudyal et al. 2024). The study conducted in a rural mining region in Central Appalachia reported that native hardwood species can significantly restore ES and ecological recovery; however, it may take several decades to reach the pre-mining stages due to degraded soil conditions (Cribari et al. 2021, 2022). Several regional and global studies report similar contexts. For instance, research in Denmark found that abandoned mine lands store significantly less carbon than the protected areas with forests (Conradi et al. 2021). In the Midwestern United States, Qiu & Turner (2013) revealed that effective carbon sequestration is constrained by historical land degradation. In the Mediterranean region, a study by Mirici and Berberoglu (2024) revealed that rural landscapes provide important climate-regulating ES; however, the unsustainable LULCC led to significant carbon losses. In Ethiopia, deforestation caused a substantial decrease in ES, mainly carbon sequestration (Demissie 2022); and in Brazil, urbanization and deforestation reduced the carbon storage at the watershed, while conservation incentives like Brazil’s watershed payment program played a critical role in maintaining these carbon sinks (Osuna et al. 2014).

The approach used in BSRB of planting native hardwood species of trees is very important in mitigating global climate change (Amichev et al. 2008; X. Li et al. 2018). Research on identifying cultivable crop species suitable for the different soil types in BSRB, running a research trial, and commercializing opportunities to the local communities and private landowners could further enhance the restoration. Moreover, incorporating carbon costs and monetizing the ES into the watershed management decisions could prove beneficial in enhancing landscape conservation and ecosystem services (Mardonova & Han 2023). For instance, ES provided by PWs and NPWs is worth a value ranging from USD 6191.3 (NPWs) to USD 6340.2 (PWs) in 2021. Such valuation can support policy planning, compensation mechanisms, and bring market-based watershed conservation solutions (Brander et al. 2024; Gao et al. 2019), including watershed ecological compensation programs like those implemented in China (Gao et al. 2019). Additionally, it can guide more sustainable land use decisions and improved investments in appropriate projects that emphasize increasing carbon sequestration (Benez-Secanho & Dwivedi 2020). The carbon credit mechanism attracts investments in carbon sequestration projects (Akrofi 2024) and carbon pricing and trading schemes in regulatory and voluntary carbon markets (World Bank Organization 2024). However, fragmented landownership, low institutional capacity, and socio-economic challenges could constrain their applicability. Therefore, the carbon credit mechanism could be presented as a supportive strategy within the larger framework of public-sector policies, reclamation programs, and coordinated watershed governance. In both PWs and NPWs, carbon-based spatial planning strategies should be focused on optimizing the conservation of vulnerable watersheds (Mirici & Berberoglu 2024). Those watersheds that are highly unstable due to LULCC and have fluctuations in carbon storage and sequestration capacity should be focused on, and the results of this study should guide the landscape management decisions in the present and future planning contexts. These ecologically recovering watersheds identified in PWs and NPWs should be protected from urbanization to sustainably balance the environment.

Although this study offers insights into the spatial and temporal dynamics of climate-regulating ecosystem services in PWs and NPWs within a coal-mined and reclaimed landscape, there are some limitations. The land use land cover maps derived from NLCD are limited to land cover classes, and NLCD does not differentiate forests based on stand age, successional stages, or species composition, which could influence carbon accumulation rates and biodiversity outcomes. The InVEST model is a standard tool for ecosystem services assessment, and it captures potential carbon storage and sequestration based on LULC configuration, not on actual biological growth. We included only 17 non-priority watersheds and all 17 priority watersheds. However, several other non-priority watersheds within BSRB lie beyond the scope of our study and, therefore, were not examined. We did not explicitly construct a no-policy or business-as-usual scenario through counterfactual modeling. Instead, the comparative analysis between priority and non-priority watersheds was purposively designed to approximate a relative baseline, where NPWs function as a proxy for business-as-usual conditions and PWs reflect active policy intervention and management focus.

Future studies could enhance carbon estimates by integrating LiDAR data, FIA plots, and species-specific datasets, improving biodiversity and carbon dynamics evaluations. Research into the trade-offs between carbon-based ecosystem services and others could identify ecologically significant regions. Additionally, examining the drivers of land-use and land-cover changes, along with socio-economic factors and vulnerability metrics at the census block group level, would support effective watershed prioritization and sustainable management. In addition to this, future studies could focus on predicting ecosystem services in the BSRB over the next five years and incorporate water-based ecosystem services mapping in those PWs and NPWs, which would provide important information for watershed managers, basin coordinators, and policymakers.

## Conclusions and Policy Implications

This study examined the dynamics of carbon storage and carbon sequestration across PWs and NPWs of the BSRB of the Appalachian mountainous region in Eastern Kentucky from 2001 to 2021. The findings indicate that carbon storage and sequestration could have been influenced by historical land use and land cover changes resulting from mining, early reclamation practices, and restoration policies following the implementation of scientific restoration approaches under ARRI. Although ecosystem services declined in the initial years between 2001 and 2006, and 2006 and 2011, the subsequent recovery was evident from 2011 to 2021 in both PWs and NPWs. The recovery of climate-regulating ecosystem services in the PWs and NPWs appears to be gradually reversing the long-term ecological degradation and carbon losses. The results revealed PWs have a relatively higher carbon sequestration rate than NPWs (Supplementary Table S11). Some watersheds are experiencing a decline in measured ecosystem services and have not recovered over the years. This study suggests that those identified watersheds should be prioritized in the planning process to ensure an equitable and sustainable development across BSRB.

Moreover, our findings offer several useful recommendations for policy implications in BSRB: (1) establishing a state-level carbon registry for coal-mined and reclaimed lands could foster transparent accounting of CS and CSE benefits and facilitate partnerships with private offset buyers; (2) underperforming NPWs should be prioritized for restoration by combining ecosystem service indicators with measures of ecological and social vulnerability; (3) institutionalizing annual ecosystem service monitoring within Kentucky Division of Water (KDOW) programs using Earth observation and geospatial tools would support continuous evaluation of restoration outcomes; (4) incorporating citizen science and youth-led restoration initiatives in vulnerable watersheds could strengthen local stewardship and improve long-term sustainability outcomes; (5) finally, the comparative PWs and NPWs models used in this study can be extended to other coal-producing Appalachian states, such as West Virginia and Pennsylvania, to support cross-state learning and coordination.

## Supplementary information

Supplementary information

## Data Availability

The datasets employed and/or examined in this study can be obtained upon reasonable request from the corresponding author.

## References

[CR1] Abbasnezhad B, Abrams JB, Wenger SJ (2024) The impact of projected land use changes on the availability of ecosystem services in the upper flint river watershed, USA. Land 13(6). 10.3390/land13060893

[CR2] Akrofi MM (2024) Green hotspots? Unveiling global hotspots and shifting trends in carbon credit projects. Sustain Dev, 1–15. 10.1002/sd.3209

[CR3] Amichev BY, Burger JA, Rodrigue JA (2008) Carbon sequestration by forests and soils on mined land in the Midwestern and Appalachian coalfields of the U.S. For Ecol Manag 256(11):1949–1959. 10.1016/j.foreco.2008.07.020.

[CR4] Atalay H (2025) Spatial autocorrelation analysis of CO and NO_2_ Related to forest fire dynamics. 2

[CR5] Atkinson G, Gundimeda H (2006) Accounting for India’s forest wealth. Ecol Econ 59:462–476. 10.1016/j.ecolecon.2005.10.022.

[CR6] Bai Y, Ochuodho TO, Yang J, Agyeman DA (2021) Bundles and hotspots of multiple ecosystem services for optimized land management in Kentucky, United States. Land 10(1):1–14. 10.3390/land10010069.

[CR7] Bai Y, Yang J, Ochuodho TO, Thapa B (2024) Impacts of land ownership and forest fragmentation on water-related ecosystem services provision, dynamics and their economic valuation in Kentucky. Land 13(7). 10.3390/land13070984

[CR8] Balasubramanian A (2016) Coal Mining Methods. 1–9. 10.13140/RG.2.2.19117.08162

[CR9] Benez-Secanho FJ, Dwivedi P (2020) Analyzing the provision of ecosystem services by conservation easements and other protected and non-protected areas in the Upper Chattahoochee Watershed. Sci Total Environ 717: 137218. 10.1016/j.scitotenv.2020.137218. 32092803 10.1016/j.scitotenv.2020.137218

[CR10] Benez-Secanho FJ, Dwivedi P, Ferreira S, Hepinstall-Cymerman J, Wenger S (2022) Trade-offs between the value of ecosystem services and connectivity among protected areas in the upper Chattahoochee Watershed. Environ Manag 69(5):937–951. 10.1007/s00267-021-01584-6. 10.1007/s00267-021-01584-6 35103811

[CR11] Brander LM, de Groot R, Schägner JP, Guisado-Goñi V, van ’t Hoff V, Solomonides S, McVittie A, Eppink F, Sposato M, Do L, Ghermandi A, Sinclair M, Thomas R (2024) Economic values for ecosystem services: A global synthesis and way forward. Ecosyst Serv 66(February). 10.1016/j.ecoser.2024.101606

[CR12] Brown SL, Schroeder P, Kern JS (1999) Spatial distribution of biomass in forests of the eastern USA. For Ecol Manag 123(1):81–90. 10.1016/S0378-1127(99)00017-1.

[CR13] Butler, P. R., Iverson, L. R., F. R. Thompson, Brandt, L. A., Handler, S. D., Janowiak, M. K., Shannon, P. D., Swanston, C., Karriker, K., Bartig, J., Connolly, S., Dijak, W. D., Bearer, S., Blatt, S. L., Brandon, A., Byers, E., Coon, C., Culbreth, T., Daly, J., … Zegre, N. (2015). *Central Appalachians Forest Ecosystem Vulnerability Assessment and Synthesis: A Report from the Central Appalachians Climate Change Response Framework Project*. *February*, 322. https://www.nrs.fs.fed.us/pubs/45688%0A

[CR14] Carey DI (2009) Big Sandy/Little Sandy and Tygarts Creek Basins. https://kgs.uky.edu/kgsweb/olops/pub/kgs/mc192_12.pdf.

[CR15] Chang X, Xing Y, Wang J, Yang H, Gong W (2022) Effects of land use and cover change (LUCC) on terrestrial carbon stocks in China between 2000 and 2018. Resour Conserv Recycling 182(March):106333. 10.1016/j.resconrec.2022.106333.

[CR16] Christopher LM, Cox JJ, Spear SF, Edwards JW, Cruz JLD, La Muller LI, Ford WM (2020) Terrestrial wildlife in the post-mined Appalachian landscape: status and opportunities. In: Zipper CE, Skousen J (eds.), Appalachia’s Coal-Mined Landscapes. Springer, Cham. p 135–166 10.1007/978-3-030-57780-3_6

[CR17] Conradi T, Henriksen MVJ, Svenning JC (2021) Global change, novel ecosystems and the ecological restoration of post-industrial areas: the case of a former brown coal mine in Søby, Denmark. Appl Veg Sci 24(3):1–12. 10.1111/avsc.12605.

[CR18] Costanza R, Arge R, Groot R, De, Farber S, Grasso M, Hannon B (1998) The value of the world’s ecosystem services and natural capital. Ecol Econ 1:3–15. 10.1016/S0921-8009(98)00020-2.

[CR19] Cribari V, Strager MP, Geneletti D, Yuill C (2022) Analyzing the interactions among multiple ecosystem services in a rural mining region in Central Appalachians. Ecosyst People 18(1):189–211. 10.1080/26395916.2022.2043445.

[CR20] Cribari V, Strager MP, Maxwell AE, Yuill C (2021) Landscape changes in the southern coalfields of West Virginia: multi-level intensity analysis and surface mining transitions in the headwaters of the Coal River from 1976 to 2016. Land 10(7). 10.3390/land10070748

[CR21] Demissie TA (2022) Land use and land cover change dynamics and its impact on watershed hydrological parameters: the case of Awetu watershed, Ethiopia. J Sediment Environ 7(1):79–94. 10.1007/s43217-021-00084-1.

[CR22] ESRI (2025) How Spatial Autocorrelation (Global Moran’s I) works. https://pro.arcgis.com/en/pro-app/latest/tool-reference/spatial-statistics/h-how-spatial-autocorrelation-moran-s-i-spatial-st.htm

[CR23] Gao X, Shen J, He W, Sun F, Zhang Z, Zhang X, Zhang C, Kong Y, An M, Yuan L, Xu X (2019) Changes in ecosystem services value and establishment of watershed ecological compensation standards. Int J Environ Res Public Health 16(16):1–30. 10.3390/ijerph16162951. 10.3390/ijerph16162951 PMC6719205 31426344

[CR24] Geneletti D, Scolozzi R, Adem Esmail B (2018) Assessing ecosystem services and biodiversity tradeoffs across agricultural landscapes in a mountain region. Int J Biodivers Sci,Ecosyst Serv Manag 14(1):188–208. 10.1080/21513732.2018.1526214.

[CR25] Greenstone M, Kopits E, Wolverton A (2013) Developing a social cost of carbon for us regulatory analysis: A methodology and interpretation. Rev Environ Econ Policy 7(1):23–46. 10.1093/reep/res015.

[CR26] Gurung K, Yang J, Fang L (2018).Assessing ecosystem services from the forestry-based reclamation of surface mined areas in the North Fork of the Kentucky River watershed. Forests 9(10). 10.3390/f9100652

[CR27] Hamel P, Guerry AD, Polasky S, Han B, Douglass JA, Hamann M, Janke B, Kuiper JJ, Levrel H, Liu H (2021) Mapping the benefits of nature in cities with the InVEST software.10.1038/s42949-021-00027-9

[CR28] Heath LS, Smith JE, Woodall CW, Azuma DL, Waddell KL (2011) Carbon stocks on forestland of the United States, with emphasis on USDA Forest Service ownership. Ecosphere 2(1). 10.1890/ES10-00126.1

[CR29] Höök M, Aleklett K (2009) Historical trends in American coal production and a possible future outlook. Int J Coal Geol 78(3):201–216. 10.1016/j.coal.2009.03.002.

[CR30] Houghton RA (2012) Historic changes in terrestrial carbon storage.In: Lal J, Lorenz R, Hüttl K, Schneider R, von Braun B (eds) Recarboniz. Springer, Dordrecht. 10.1007/978-94-007-4159-1_4

[CR31] IPCC (2006) IPCC Methodology Forest Land. Agric For Land Use, 4:83

[CR32] Jerath M, Bhat M, Rivera-Monroy VH, Castañeda-Moya E, Simard M, Twilley RR (2016) The role of economic, policy, and ecological factors in estimating the value of carbon stocks in Everglades mangrove forests, South Florida, USA. Environ Sci Policy 66:160–169. 10.1016/j.envsci.2016.09.005.

[CR33] Kandel S, Gyawali B, Shrestha S, Zourarakis D, Antonious G, Gebremedhin M, Pokhrel B (2023) Estimation of runoff and sediment yield in response to temporal land cover change in Kentucky, USA. Land 12(1). 10.3390/land12010147

[CR34] Li L, Yang Y, Cui T, Li R, Zheng H (2023) Land use, climate, and socioeconomic factors determine the variation in hydrologic-related ecosystem services in the ecological conservation zone, Beijing, China. Water, 15(11) 10.3390/w15112022

[CR35] Li X, Stainback A, Barton C, Yang J (2018) Valuing the environmental benefits from reforestation on reclaimed surface mines in Appalachia. J Am Soc Min Reclam 7(1):1–29. 10.21000/jasmr180100029.

[CR36] Li Z, Li X, Wang Y, Ma A, Wang J (2004) Land-use change analysis in Yulin prefecture, northwestern China using remote sensing and GIS. Int J Remote Sens 25(24):5691–5703. 10.1080/01431160412331291206.

[CR37] Lindberg TT, Bernhardt ES, Bier R, Helton AM, Brittany Merola R, Vengosh A, Di Giulio RT (2011) Cumulative impacts of mountaintop mining on an Appalachian watershed. Proc Natl Acad Sci USA 108(52):20929–20934. 10.1073/pnas.1112381108. 22160676 10.1073/pnas.1112381108 PMC3248525

[CR38] Liu C, Zhang L, Li F, Jin X (2014) Spatial modeling of the carbon stock of forest trees in Heilongjiang Province, China. J For Res 25(2):269–280. 10.1007/s11676-014-0458-x.

[CR39] Liu J, Liu S, Loveland TR (2006) Temporal evolution of carbon budgets of the Appalachian forests in the U.S. from 1972 to 2000. For Ecol Manag 222(1–3):191–201. 10.1016/j.foreco.2005.09.028.

[CR40] Ma J, Hao Z, Shen Y, Zhen Z (2025) Spatial-temporal evolution of carbon storage and its driving factors in the Shanxi section of the Yellow River Basin, China. Ecol Model 502(January):111039. 10.1016/j.ecolmodel.2025.111039.

[CR41] Mardonova M, Han YS (2023) Environmental, hydrological, and social impacts of coal and nonmetal minerals mining operations. J Environ Manag 332(February):117387. 10.1016/j.jenvman.2023.117387. 10.1016/j.jenvman.2023.117387 36736087

[CR42] Millenium Ecosystem Assessment (2005) Ecosystems and Human Well-being: Synthesis. In Island Press. https://www.millenniumassessment.org/documents/document.356.aspx.pdf.

[CR43] Min X, Zhang B, Wang Y (2025) Mining activities drive the temporal and spatial changes of ecosystem carbon storage in coal resource-based city with high groundwater table. Habitat Int 161 10.1016/j.habitatint.2025.103420

[CR44] Mirici ME, Berberoglu S (2024) Terrestrial carbon dynamics and economic valuation of ecosystem service for land use management in the Mediterranean region. Ecol Inform 81(June 2023):102570. 10.1016/j.ecoinf.2024.102570.

[CR45] Mishra P, Pandey CM, Singh U, Gupta A, Sahu C, Keshri A (2019) Descriptive statistics and normality tests for statistical data. Ann Card Anaesth 22(1):67–72. 10.4103/aca.ACA_157_18. 30648682 10.4103/aca.ACA_157_18 PMC6350423

[CR46] Moore R, Williams T, Rodriguez E, Hepinstall C (2011) Quantifying the value of non-timber ecosystem services from Georgia’s private forests. Georgia Forestry Foundation 51. http://gfagrow.org/wp-content/uploads/2015/11/Ecosystem-Services-Final-Report.pdf

[CR47] Muga G, Tiando DS, Liu C (2025) Spatial relationship between carbon emissions and ecosystem service value based on land use: a case study of the Yellow River Basin. PLoS ONE 20(2 February):1–20. 10.1371/journal.pone.0318855. 10.1371/journal.pone.0318855 PMC11845033 39982886

[CR48] Olson JS, Watts JA, Allison LJ (1985) Major world ecosystem complexes ranked by carbon in live vegetation: a database. Sciences 5862. http://www.osti.gov/energycitations/product.biblio.jsp?osti_id=6944260

[CR49] Osuna VR, Börner J, Nehren U, Prado RB, Gaese H, Heinrich J (2014) Priority areas for watershed service conservation in the Guapi-Macacu region of Rio de Janeiro, Atlantic Forest, Brazil. Ecol Process 3(1):1–21. 10.1186/s13717-014-0016-7.

[CR50] Pandeya S, Gyawali BR, Upadhaya S, Zourarakis D, Gebremedhin M (2025) Patterns of change: Land use/land cover dynamics in priority vs. non-Priority watersheds in Eastern Kentucky. Environ. Challenges 20:101264. 10.1016/j.envc.2025.101264.

[CR51] Poudyal NC, Gyawali BR, Acharya S (2024) Reclamation satisfaction and post-mining land use potential in Central Appalachia, US. Extract Ind Soc 20(September):101550. 10.1016/j.exis.2024.101550.

[CR52] Qiu J, Turner MG (2013) Spatial interactions among ecosystem services in an urbanizing agricultural watershed. Proc Natl Acad Sci USA 110(29):12149–12154. 10.1073/pnas.1310539110. 23818612 10.1073/pnas.1310539110 PMC3718150

[CR53] Rosenberger RS, Loomis J (2003) Benefit transfer. A Primer on Nonmarket Valuation, 445–482. 10.1007/978-94-007-0826-6_12

[CR54] Sharp R, Tallis H, Ricketts T, Guerry A, Wood SA, Chaplin-Kramer R, Nelson E, Ennaanay D, Wolny S, Olwero N (2014) InVEST User’s Guide. The Natural Capital Project: Stanford, CA, USA, 306

[CR55] Shobairi O, Beirami BA, Hemmati S, Pirbasti MA (2024) Carbon storage in Hunan province: monitoring, modeling, and management strategies for climate change mitigation. Archiv Photogramm Cartogr Remote Sensing, 36(ISSN 2083-2214, eISSN 2391-9477), 11–32. 10.14681/apcrs-2023-001

[CR56] Silver WL, Ryals R, Eviner V (2010) Soil carbon pools in California’s annual grassland ecosystems. Rangel Ecol Manag 63(1):128–136. 10.2111/REM-D-09-00106.1.

[CR57] Sun B, Du J, Chong F, Li L, Zhu X, Zhai G, Song Z, Mao J (2023) Spatio-temporal variation and prediction of carbon storage in terrestrial ecosystems in the Yellow River basin. Remote Sens 15(15). 10.3390/rs15153866

[CR58] Timilsina N, Escobedo FJ, Cropper WP, Abd-Elrahman A, Brandeis TJ, Delphin S, Lambert S (2013) A framework for identifying carbon hotspots and forest management drivers. J Environ Manag 114:293–302. 10.1016/j.jenvman.2012.10.020. 10.1016/j.jenvman.2012.10.020 23171606

[CR59] U.S. Environmental Protection Agency (2024) Ecosystem services research. https://www.epa.gov/eco-research/ecosystem-services-research

[CR60] Waleed M, Sajjad M, Shazil MS (2024) Urbanization-led land cover change impacts terrestrial carbon storage capacity: a high-resolution remote sensing-based nation-wide assessment in Pakistan (1990–2020). Environ Impact Assess Rev 105(December 2023):107396. 10.1016/j.eiar.2023.107396.

[CR61] Wang H, Wu L, Yue Y, Jin Y, Zhang B (2024) Impacts of climate and land use change on terrestrial carbon storage: a multi-scenario case study in the Yellow River Basin (1992–2050). Sci Total Environ 930(April). 10.1016/j.scitotenv.2024.172557 10.1016/j.scitotenv.2024.172557 38643873

[CR62] Were K, Singh BR, Dick ØB (2016) Spatially distributed modelling and mapping of soil organic carbon and total nitrogen stocks in the Eastern Mau Forest Reserve, Kenya. J Geogr Sci 26(1):102–124. 10.1007/s11442-016-1257-4.

[CR63] Wickham JD, Wade TG, Norton DJ (2014) Spatial patterns of watershed impervious cover relative to stream location. Ecol Indic 40:109–116. 10.1016/j.ecolind.2014.01.013.

[CR64] World Bank Organization (2024) Global carbon pricing revenues top a record $100 billion. World Bank Group

[CR65] Xie Q, Han Y, Zhang L, Han Z (2023) Dynamic evolution of land use/land cover and its socioeconomic driving forces in Wuhan, China. Int J Environ Res Public Health 20(4). 10.3390/ijerph20043316 10.3390/ijerph20043316 PMC9961176 36834009

[CR66] Yang S, Li L, Zhu R, Luo C, Lu X, Sun M, Xu B (2024) Assessing land-use changes and carbon storage: a case study of the Jialing River Basin, China. Sci Rep 14(1):1–18. 10.1038/s41598-024-66742-2. 38987401 10.1038/s41598-024-66742-2 PMC11237025

[CR67] Yang X, Wang K, Zhang Y (2024) Spatial spillover effects of urbanization on ecosystem services under altitude gradient. Land 13(5). 10.3390/land13050622

[CR68] Zégre NP, Maxwell A, Lamont S (2013) Characterizing streamflow response of a mountaintop-mined watershed to changing land use. Appl Geogr 39(March 2020):5–15. 10.1016/j.apgeog.2012.11.008.

