# Supplementary material for: From Coalfields to Carbon Sinks: Examining the Policy Effects on the Dynamics of Ecosystem Services in the Watersheds of Eastern Kentucky, USA
Source: Environ Manage. 2026 Apr 9;76(5):141. doi: 10.1007/s00267-026-02431-2 (PMC13065603; doi:10.1007/s00267-026-02431-2)
Supplement: Supplementary file 1 — Supplementary information [file 267_2026_2431_MOESM1_ESM.docx]

**From Coalfields to Carbon Sinks: Examining the Dynamics of Ecosystem Services in the Watersheds of Eastern Kentucky, USA, and their Policy Implications**

Shreesha Pandeya^1^*, Buddhi Gyawali^1^, Suraj Upadhaya^1^, Maheteme Gebremedhin^1^, Demetrio Zourarakis^2^

^1^College of Agriculture, Health and Natural Resources, Kentucky State University, Frankfort, KY, 40601, USA

^2^Martin-Gatton College of Agriculture, Food and Environment, University of Kentucky, Lexington, KY, 40546, USA

*Corresponding Author:

Shreesha Pandeya

School of Agriculture and Natural Resources

College of Agriculture, Health and Natural Resources

Kentucky State University, Frankfort, KY 40601

Email : shreesha.pandeya1@kysu.edu


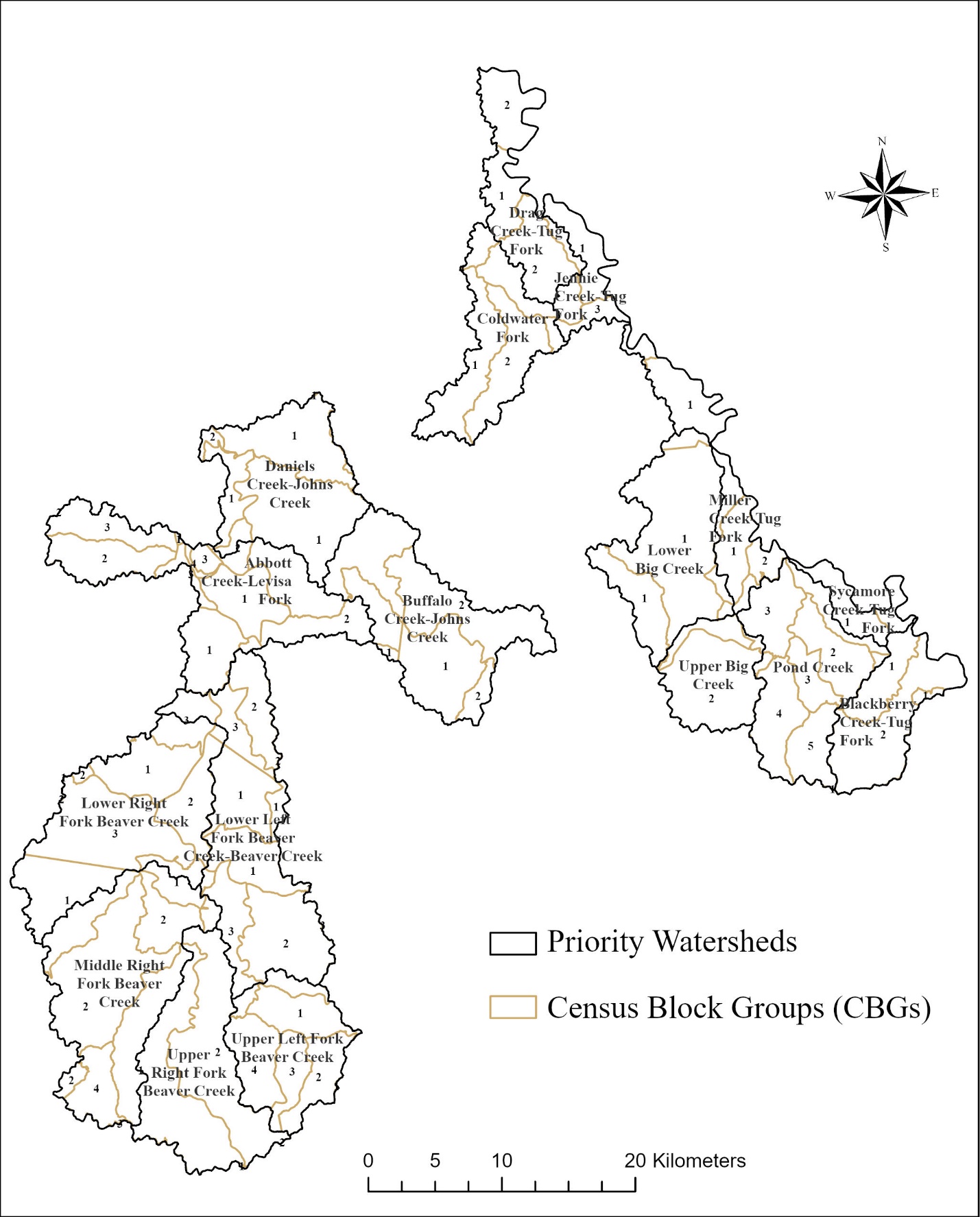


Figure S1. Priority watersheds at HUC-12 of the Big Sandy River Basin.


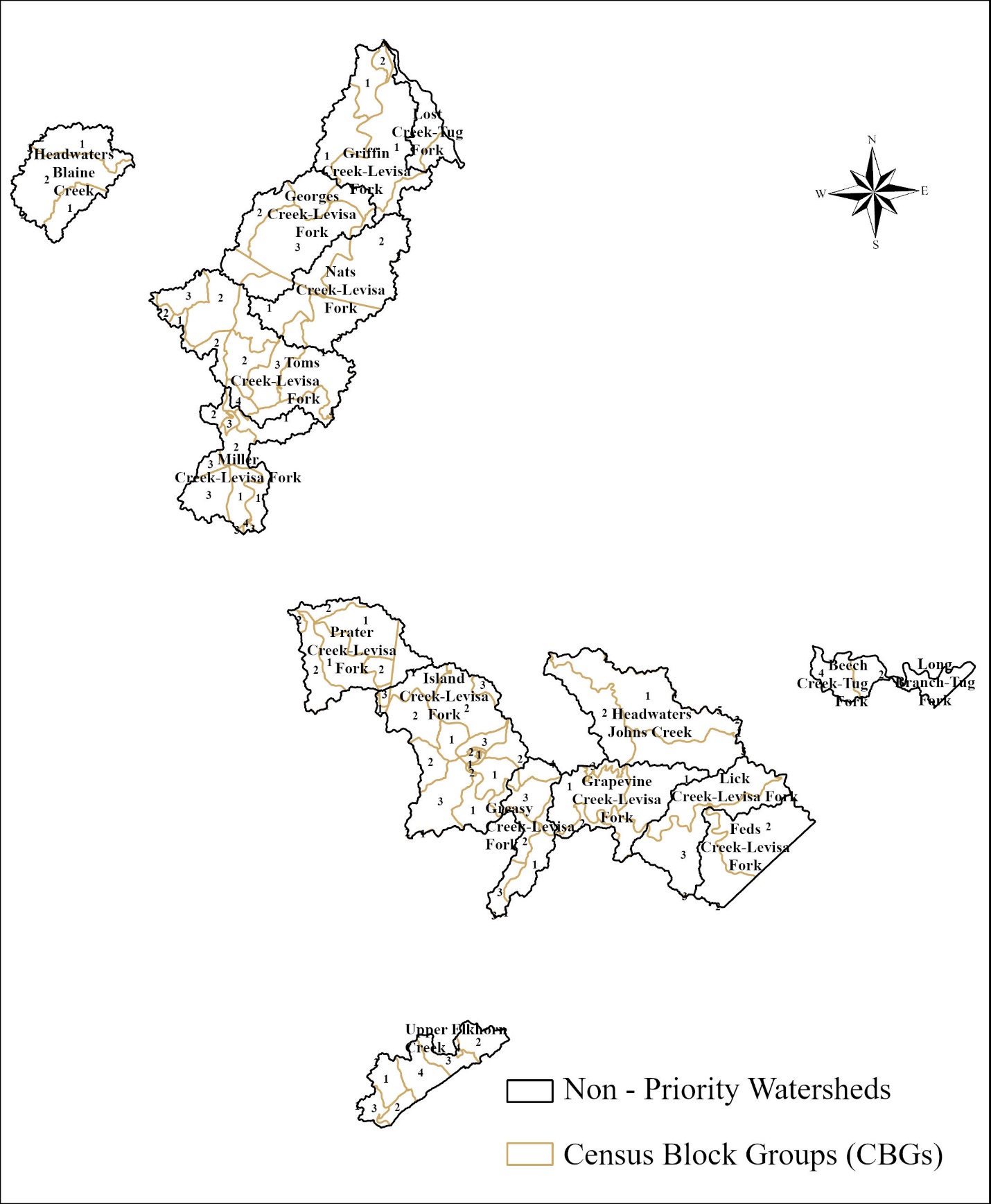


Figure S2: Non-priority watersheds at HUC-12 of the Big Sandy River Basin.

Table S1: Carbon pool coefficients used for the InVEST model.

| LULC | Aboveground Carbon (Mg C/ ha) | Belowground (Mg C/ ha) | Soil OM | Dead OM |
| --- | --- | --- | --- | --- |
| Water | 0.00 | 0.00 | 0.00 | 0.00 |
| Developed | 5.06 | 2.10 | 31.13 | 1.50 |
| Barren | 0.00 | 0.00 | 2.50 | 0.00 |
| Forest | 118.51 | 57.00 | 72.39 | 22.18 |
| Shrubland | 20.42 | 12.52 | 69.58 | 7.92 |
| Herbaceous(grassland) | 21.64 | 13.38 | 64.58 | 8.21 |
| Pasture and cultivated | 28.96 | 15.74 | 57.14 | 10.21 |
| Wetlands | 117.40 | 25.72 | 168.74 | 25.00 |

(Abbasnezhad et al., 2024; Amichev et al., 2008; Bai et al., 2021, 2024; Benez-Secanho et al., 2022; Benez-Secanho & Dwivedi, 2020; Brown et al., 1999; Geneletti et al., 2018; Gurung et al., 2018; Heath et al., 2011; IPCC, 2006; Jerath et al., 2016; X. Li et al., 2018; Olson et al., 1985; Qiu & Turner, 2013; Sharp et al., 2014; Silver et al., 2010; Timilsina et al., 2013; Waleed et al., 2024).

Table S2: Total Carbon Storage values (Mg C) from 2001 to 2021 in PWs and NPWs as derived from the InVEST model

| Year | Total Stored Carbon (Mg C) in PWs | Total Economic Value in PWs (million USD) | Total Carbon Stored (Mg C) in NPWs | Total Economic Value in NPWs (million USD) |
| --- | --- | --- | --- | --- |
| 2001 | 37.25 × 10⁶ | 968.58 | 33.94 × 10⁶ | 882.41 |
| 2006 | 36.81 × 10⁶ | 956.94 | 33.48 × 10⁶ | 870.38 |
| 2011 | 36.73 × 10⁶ | 954.86 | 33.40 × 10⁶ | 868.38 |
| 2016 | 36.97 × 10⁶ | 961.14 | 33.54 × 10⁶ | 871.95 |
| 2021 | 37.60 × 10⁶ | 977.62 | 33.99 × 10⁶ | 883.81 |

Table S3: Relative Change in carbon storage (Mg C/ha) in PWs and NPWs from 2001 to 2021.

| Year | PWs (%) | NPWs (%) |
| --- | --- | --- |
| 2001-2006 | -1.20 | -1.36 |
| 2006-2011 | -0.22 | -0.23 |
| 2011-2016 | 0.66 | 0.41 |
| 2016-2021 | 1.71 | 1.36 |
| 2001-2021 | 0.93 | 0.16 |

Table S4: Mann-Whitney U test results comparing watershed-level carbon storage (Mg C/ha) between PWs (N=17) and NPWs (N=17).

| Year | Median Carbon Storage in PWs (Mg C/ha) | Median Carbon Storage in NPWs (Mg C/ha) | U-value | p-value |
| --- | --- | --- | --- | --- |
| 2001 | 239.84 | 230.19 | 157.0 | 0.67 |
| 2006 | 236.42 | 227.42 | 158.0 | 0.65 |
| 2011 | 234.71 | 225.64 | 157.0 | 0.67 |
| 2016 | 236.47 | 227.55 | 166.0 | 0.46 |
| 2021 | 239.22 | 236.14 | 178.0 | 0.25 |

Table S5. Parameter-range uncertainty analysis in total carbon storage (2001 and 2021).

| Watershed Type | Year | Low  (Mg C) | Mean  (Mg C) | High  (Mg C) | Error Margin  (Mg C) |
| --- | --- | --- | --- | --- | --- |
| PWs | 2001 | 8.53×10⁶ | 42.68×10⁶ | 76.84×10⁶ | 34.15×10⁶ |
| PWs | 2021 | 8.67×10⁶ | 43.03×10⁶ | 77.39×10⁶ | 34.36×10⁶ |
| NPWs | 2001 | 7.80×10⁶ | 38.93×10⁶ | 70.04×10⁶ | 31.13×10⁶ |
| NPWs | 2021 | 7.87×10⁶ | 38.395×10⁶ | 70.04×10⁶ | 31.08×10⁶ |

Table S6: Total Carbon Sequestration values (Mg C) from 2001 to 2021 in PWs and NPWs

| Year | Total Sequestered Carbon (Mg C) in PWs | Economic Value in PWs (million USD) | Total Sequestered Carbon (Mg C) in NPWs | Economic Value in PWs (million USD) |
| --- | --- | --- | --- | --- |
| 2001-2021 | 0.35 × 10⁶ | 5.49 | 0.05 × 10⁶ | 0.85 |
| 2001-2006 | -0.45 × 10⁶ | -10.38 | -0.46 × 10⁶ | -10.73 |
| 2006-2011 | -0.08 × 10⁶ | -1.86 | -0.07 × 10⁶ | -1.78 |
| 2011-2016 | 0.24 × 10⁶ | 5.61 | 0.13 × 10⁶ | 3.18 |
| 2016-2021 | 0.63 × 10⁶ | 14.71 | 0.45 × 10⁶ | 10.60 |

Table S7: Mann-Whitney U test results comparing watershed-level carbon sequestration (Mg C/ha) between PWs (N=17) and NPWs (N=17).

| Year Interval | Median Carbon Sequestration in PWs (Mg C/ha) | Median Carbon Sequestration in NPWs (Mg C/ha) | U-value | p-value |
| --- | --- | --- | --- | --- |
| 2001-2006 | -1.34 | -2.66 | 160.0 | 0.60 |
| 2006-2011 | -0.07 | -0.39 | 162.0 | 0.55 |
| 2011-2016 | 1.18 | 0.35 | 182.0 | 0.20 |
| 2016-2021 | 2.43 | 2.75 | 161.0 | 0.58 |
| 2001-2021 | 0.36 | -0.64 | 185.0 | 0.16 |

Table S8: Global Moran’s I values of Carbon Storage (Mg/ha) for PWs and NPWs in the BSRB.

| Watershed Type | Year | Morans I index | z | p |
| --- | --- | --- | --- | --- |
| NPWs | 2001 | 0.207 | 2.77 | 0.005 |
|  | 2006 | 0.22 | 2.94 | 0.003 |
|  | 2011 | 0.21 | 2.92 | 0.003 |
|  | 2016 | 0.22 | 2.91 | 0.003 |
|  | 2021 | 0.19 | 2.70 | 0.006 |
| PWs | 2001 | 0.19 | 0.71 | 0.006 |
|  | 2006 | 0.19 | 2.62 | 0.008 |
|  | 2011 | 0.18 | 2.55 | 0.01 |
|  | 2016 | 0.18 | 2.59 | 0.009 |
|  | 2021 | 0.19 | 2.70 | 0.006 |

Table S9: Global Moran’s I values of Carbon sequestration (Mg/ha) for PWs and NPWs in the BSRB

| Watershed Type | Year | Moran’s I index | z | p |
| --- | --- | --- | --- | --- |
| NPWs | 2001 - 2021 | 0.11 | 1.78 | 0.07 |
|  | 2001 - 2006 | 0.18 | 2.63 | 0.008 |
|  | 2006 - 2011 | 0.01 | 0.33 | 0.74 |
|  | 2011 - 2016 | 0.05 | 1.33 | 0.18 |
|  | 2016 - 2021 | 0.08 | 1.47 | 0.15 |
| PWs | 2001 - 2021 | 0.04 | 1.28 | 0.197 |
|  | 2001 - 2006 | 0.09 | 2.47 | 0.013 |
|  | 2006 - 2011 | -0.02 | -0.149 | 0.88 |
|  | 2011 - 2016 | 0.05 | 1.66 | 0.096 |
|  | 2016 - 2021 | 0.08 | 1.42 | 0.15 |

Table S10: Local Moran’s I values of Carbon sequestration (Mg/ha) for PWs and NPWs in the BSRB

| Watershed Type | Year | Local Moran’s I index |
| --- | --- | --- |
| NPWs | 2001 - 2021 | 0.11 |
|  | 2001 - 2006 | 0.18 |
|  | 2006 - 2011 | 0.013 |
|  | 2011 - 2016 | 0.051 |
|  | 2016 - 2021 | 0.087 |
| PWs | 2001 - 2021 | 0.12 |
|  | 2001 - 2006 | 0.14 |
|  | 2006 - 2011 | -0.06 |
|  | 2011 - 2016 | 0.13 |
|  | 2016 - 2021 | 0.18 |

Table S 11: Integrated summary of CS, CSE, EV, and Local Moran’s I results in PWs and NPWs

| Watershed Type | CS Trend | CSE Trend | Economic value (USD/ha/yr) | Local Moran’s I |
| --- | --- | --- | --- | --- |
| PWs | Increasing (2016-2021) | Recovery after 2011 | Higher | More H-H dominance |
| NPWs | Slower rate | More variable | Lower | More L-L dominance |
